# Optimizing the prescription isodose level in stereotactic volumetric-modulated arc radiotherapy of lung lesions as a potential for dose de-escalation

**DOI:** 10.1186/s13014-018-0965-6

**Published:** 2018-02-09

**Authors:** Mark Chan, Matthew Wong, Ronnie Leung, Steven Cheung, Oliver Blanck

**Affiliations:** 1 University Medical Center Schleswig–Holstein, Campus Kiel, Department for Radiation Oncology, Arnold–Heller–Straße 3, Haus 50, Karl–Lennert–Krebscentrum Nord, 24105 Kiel, Germany; 2 Imperial College London Healthcare NHS Trust, Department of Radiation Physics, London, UK; 3 Tuen Mun Hospital, Department of Clinical Oncology, Special Administrative Region of China, Hong Kong, Hong Kong, Special Administrative Region of China; 4 Saphir Radiosurgery Center Northern Germany, Güstrow, Germany

## Abstract

**Background:**

To derive and exploit the optimal prescription isodose level (PIL) in inverse optimization of volumetric modulated arc radiotherapy (VMAT) as a potential approach to dose de–escalation in stereotactic body radiotherapy for non–small cell lung carcinomas (NSCLC).

**Methods:**

For ten patients, inverse Monte Carlo dose optimization was performed to cover 95% PTV by varying prescription isodose lines (PIL) at 60 to 80% and reference 85%. Subsequently, these were re–normalized to the median gross tumor volume dose (GTV–based prescription) to assess the impacts of PTV and normal tissue dose reduction.

**Results:**

With PTV–based prescription, GTV mean dose was much higher with the optimized PIL at 60% with significant reduction of normal lung receiving 30 to 10 Gy (*V*_*30–10Gy*_), and observable but insignificant dose reduction to spinal cord, esophagus, ribs, and others compared with 85% PIL. Mean doses to the normal lung between PTV and GTV was higher with 60–70% PIL than 85%. The dose gradient index was 5.0 ± 1.1 and 6.1 ± 1.4 for 60 and 85% PIL (*p* < 0.05), respectively. Compared with the reference 85% PIL plan using PTV–base prescription, significant decreases of all normal tissue doses were observed with 60% and 70% PIL by GTV–based prescription. Yet, the resulting biological effective (BED) mean doses of PTV remain sufficiently high, ranging 104.2 to 116.9 Gy _*α/β* = 10_.

**Conclusions:**

Optimizing the PIL with VMAT has notable advantage of improving the dosimetric quality of lung SBRT and offers the potential of dose de–escalation for surrounding tissues while increasing the GTV dose simultaneously. The clinical implication of re–normalizing plans from PTV–prescription at 60–70% to the GTV median dose requires further investigations.

## Background

Optimization of stereotactic body radiotherapy (SBRT) plan quality is crucial to minimize normal tissue dose and hence toxicities for inoperable early stage non–small–cell lung cancer (NSCLC). In lung SBRT small photon fields are widely used, which are known to introduce significant lateral electronic disequilibrium (LED) in heterogeneous tissue caused by out–scattering electrons not being compensated by in–scattering electrons [1]. Using the LED phenomenon for optimizing lung SBRT (LED–SBRT), which is based on the differential reductions of lung and tumor doses caused by the LED in order to steepen the dose gradients and thereby increasing the dose within the tumor while reducing the dose in the normal lung, has been recently proposed by Disher et al. [1]. However, in order for LED–SBRT to be robust against dosimetric errors, Monte Carlo (MC) simulation techniques [2] must be employed to accurately model the particle transport.

Besides direct MC simulation, the LED phenomenon was also investigated implicitly through the relationship with the prescription isodose line (PIL) by comparing the dose results from type–A pencil beam (PB), which cannot model the LED, and type–B MC based dose calculations [3]. Using multiple dynamic conformal arcs (DCA), Oku et al. showed a clear dependence of the PIL on the dosimetric plan quality [4]. They found higher lung dose and lower dose conformity at 50% downwards to 20% PIL after the optimal improvement at 60% PIL with respect to the reference of 80% PIL. Similar results of optimal PIL at ~ 60–70% and much lower at ~ 40–50% were found for forward planned linac–based and inverse planned robotic–based SBRT lung treatments, respectively [5].

The clinical implication of lowering the PIL to 60% vs. 80% PIL in 5–fraction lung SBRT was initially studied by Takeda et al., also using DCA techniques [6]. After 6 months post–SBRT, they found no local recurrences and only a limited number of incidents of radiation pneumonitis ≥ Grade 2 (1 out of 15 patients) with lower PIL. Furthermore, Guckenberger et al. found in their retrospective large–scale multi–center analysis with low PIL (< 80%) a significant higher freedom from local progression as compared to higher PIL (86.8% vs. 69.1%, *p* = 0.005), again with lower toxicity for lower PIL [7]. Further evidence of the clinical effects of varying PIL was supported by a number of recent published series from European and Japanese SBRT working groups that demonstrated the iso–effectiveness of SBRT treatments between those prescribing the biological effective dose (BED) > 100 Gy_10_ (*α / β* = 10 Gy for NSCLC) to the isocenter, to the PTV periphery with 95% coverage (*D*_95%_), and to the GTV mean or median dose [8–13].

Despite the similar local control rates reported based on different prescription concepts, the inconsistency of the conventional PTV prescription concept has been well acknowledged in the latest published International Commission on Radiation Units and Measurements (ICRU) report 91 [14], pointing at increased variability of the internal GTV dose for lung SBRT. Although the report hinted at a possible solution by using a GTV–based prescription and a few other studies [15–17] coherently showed more consistent GTV dose when re–normalizing or prescribing the treatment dose to the GTV mean or median dose or *D*_99%_, the implications of such GTV–based re–normalization/re–prescription with respect to the PIL in the conventional PTV prescription concept has never been studied.

In this work, we focused on the technical feasibility of enhancing the dosimetric quality of inverse VMAT optimization by assessing the optimal PIL and with that the potential for dose de–escalation. Further on the hypothesis that the potential dependence of plan quality on the PIL is related to the LED, retrospective PB re-calculations were performed for all direct MC–optimized VMAT plans to assess the relationship of the PIL with the dosimetric changes. Ultimately, we tried to investigate the GTV–based re–normalization / re–prescription concept with respect to the PIL for VMAT LED–SBRT.

## Methods

### Patient selections and 4DCT imaging

This retrospective dosimetric study, approved by the local clinical and research ethics committee, included ten patients with primary NSCLC. The tumor size ranged from 11.7 to 82.6 cm^3^. Each patient had a four–dimensional computed tomography (4DCT) scan that was sorted into ten 3DCT image datasets with equal time share. Each 3DCT had a slice thickness of 2 mm in the axial direction. We determined the mid–ventilation (MidV) phase from the 4DCT series for treatment planning as described previously [18].

### Treatment planning and dose prescription

The GTV was contoured based on standard international guidelines and was expanded according to the mid–ventilation PTV concept [19]. Treatment prescription was 54 Gy in 3 fractions regardless of tumor location and size.

Dose constraints for the target and other OARs were defined following the guidelines in the ROSEL [20] and RTOG 0236 [21, 22] trials. It was demanded that dose to 95% of the PTV (PTV *D*_*95%*_) and 98% of the GTV (GTV *D*_*98%*_) must at least receive the prescription dose. Also the dose to 98% of PTV (PTV *D*_*98%*_) had to exceed 90% of the prescription dose. Furthermore, we limited the maximum dose in the PTV to be within 167% of the prescription dose despite the fact that the optimal dose gradient that may occur at as low as ~ 30% PIL [1] because very limited clinical trials reporting safety and toxicities with PIL < 60% were available. Dose limits of chest wall and rib may be exceeded in case of large overlap with the PTV, as suggested in the RTOG 0915 trial [23].

### VMAT-based PIL-SBRT optimization

For all patients, volumetric–modulated arc radiotherapy (VMAT) optimizations of lung SBRT plans were performed using the Monaco treatment planning system (TPS, v.5.0, Elekta, Sweden) for the Elekta Agility™ linear accelerator (linac) equipped with a multileaf collimator (MLC) with 160 leaf pairs of 5 mm width. The VMAT plans consisted of two either full or partial 6 Mega–voltage (MV) coplanar arcs, depending on the location of the tumor to avoid direct beam entrance to the contralateral lung and other central organs at risk (OAR).

For each patient, a reference plan at 85% PIL was generated while keeping the dose constraints of the OARs as low as reasonably achievable (ALARA). Unlike the strategy with DCA, where the beam penumbra margin is manually adjusted to achieve varying levels of PIL, the MLC shapes in VMAT are completely controlled by the optimizer in achieving the dose–volume histogram (DVH) objectives. To implicitly manipulate the PIL in the inverse optimization process, we varied the maximum dose to the PTV and the GTV together with demanding higher minimum dose or DVH constraints to the GTV to achieve a lower or higher PIL. Dose constraints of all other OAR were kept unchanged in all inverse optimized plans for each patient to minimize the planner–related bias on the final plan quality as much as possible.

Using these optimization strategies, four plans were generated, with the reference corresponding to 85% PIL, and the other three corresponding to 80%, 70% and 60% PILs. All VMAT plans were directly optimized by Monte Carlo (MC) simulation technique [24] and the final dose distributions were calculated to dose to medium in medium (*D*_*m,m*_) using 2 mm dose grid and 0.5% relative statistical uncertainty.

### Plan evaluation and statistical analysis

Dosimetric parameters of the reference plan and the PIL–optimized plans as defined in ROSEL [20] and RTOG 0236 [21, 22] trial protocols were compared, which included the normalized volume received at least 5 to 30 Gy (*V*_5–30Gy_) by the normal lungs, the absolute volume received at least 30 Gy and 100% of the prescription dose by the chest wall, dose to 1% (*D*_1%_) of the cord, trachea, bronchus, esophagus and heart. The near-minimum dose *D*_98%_ to PTV and GTV mean dose (*D*_mean_) were also included. The target dose conformity was assessed using the Paddick’s conformity index [25]. The dose gradient was evaluated by the ratio of 50% prescription isodose volume to the PTV (*R*_*50%*_), and its constraint was adapted according to Xiao et al. [22] based on their retrospective re–evaluation of the dosimetric effects of heterogeneity corrections for the case submissions in the RTOG 0236 trial [21].

Further on the hypothesis that the potential dependence of plan quality on the PIL is related to the LED, all the direct MC–optimized plans were re–calculated by a PB dose engine developed by Jelen et al. [26] The resulting difference was evaluated based on mean dose and *D*_2%_ of the PTV border (i.e., PTV minus GTV) because it coincided with the low density tissue and the field edge where the LED was likely most severe.

Following the GTV–based prescription approach described by Bibault et al. [8] and Muira et al. [16], all original PTV–based prescription plans of varying PILs were re–normalized such that the GTV *D*_*50%*_ equals 54 Gy. The resultant GTV–prescribed plans at the optimal 60% and 70% PILs were compared to the reference PTV–based prescription plans at 85% PIL to study the implications of varying PILs in different prescription concepts.

Statistical comparisons of various plan quality parameters between plans of different PILs were performed by Freidman’s test. When statistical significance was found, further *post–hoc* test by the default Tukey’s honest significant difference criterion was performed using the Matlab statistics toolbox (MathWorks, Natick, MA). Further, Wilcoxon’s test was performed to assess the significance of normal tissue dose reductions achieved with GTV–based prescription compared with the reference 85% PIL plan with PTV–based prescription. Test results were considered statically significant at *p* < 0.05.

## Results

### VMAT-based PIL-SBRT optimization

GTV mean doses (*D*_*mean*_) increased from 85% PIL by 6.1 ± 2.2% (mean ± 1 standard deviation (SD)), 19.2 ± 3.3%, and 35.0 ± 5.5% with decreasing PILs to 80%, 70% and 60%, respectively (*p* < 0.01). The mean near–minimum dose *D*_*98%*_ of PTV showed an observable but insignificant descending trend with decreasing PIL and the differences among PILs of individual patients were within 2%.

**Fig. 1:**
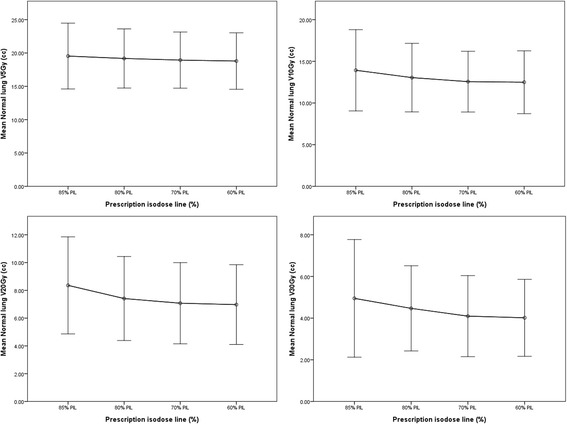
Variations of averaged normal lung volumes receiving at least 5, 10, 20 and 30 Gy (i.e., *V*_*5Gy*_, *V*_*10Gy*_, *V*_*20Gy*_, and *V*_*30Gy*_) as a function of prescription isodose levels. Error bars represent one standard deviations of *V*_*5Gy*_, *V*_*10Gy*_, *V*_*20Gy*_, and *V*_*30Gy*_

**Fig. 2:**
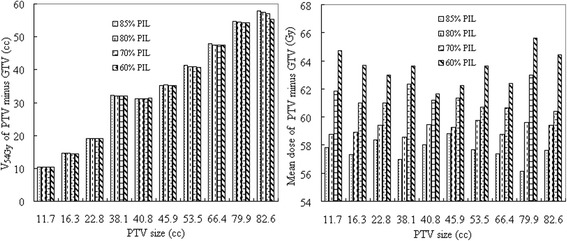
(Left) shows the normal lung volume between the planning target volume (PTV) and the gross tumor volume (GTV) receiving at least 54 Gy (V_54Gy_), and (right) the mean dose to this volume for individual lesions. Lesions are sorted according to size

**Fig. 3:**
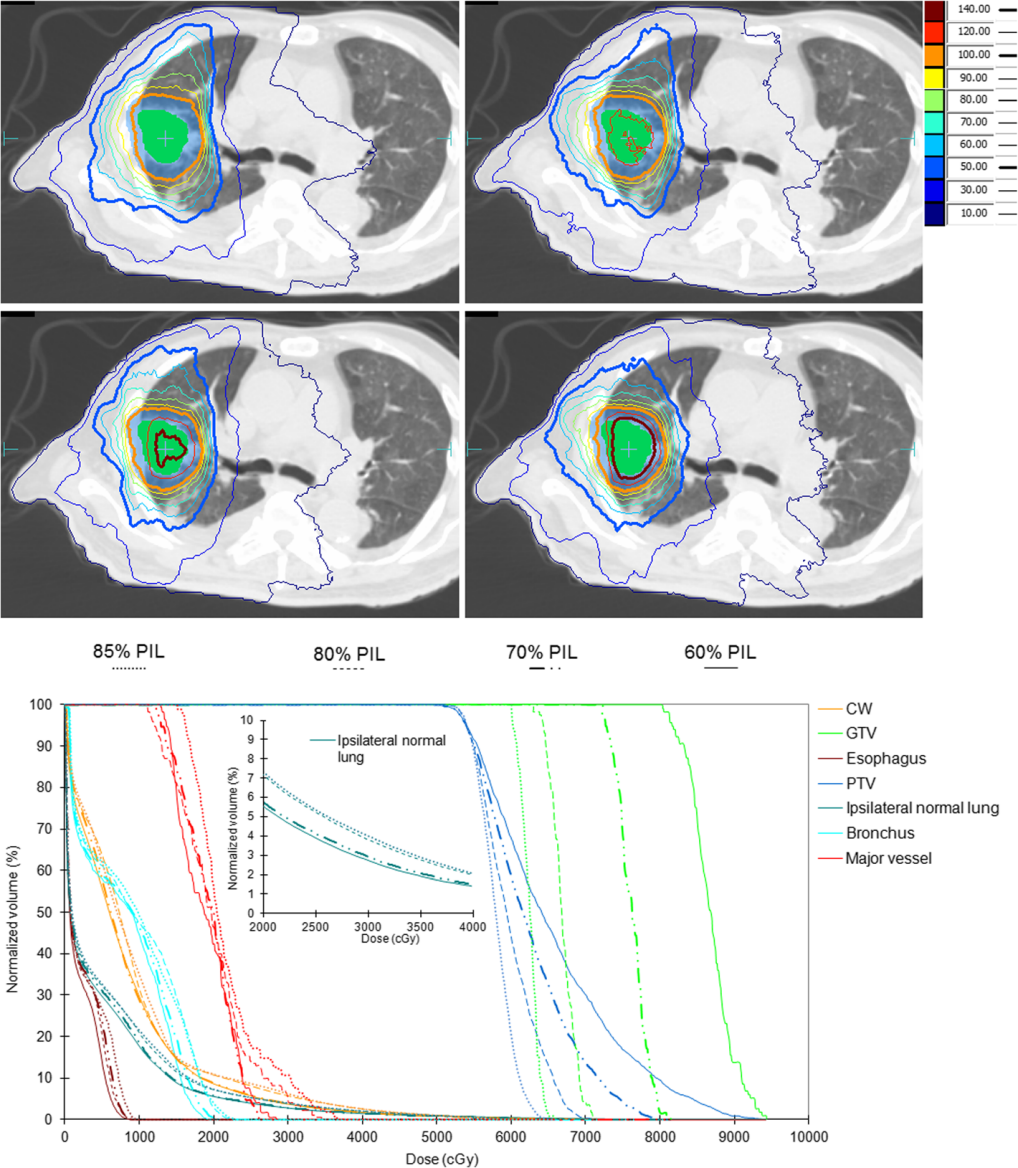
Example dose distributions achieved with varying PIL–optimized VMAT plans in the axial plane and the dose–volume histograms (DVH) of selected organs. The PILs are displayed with 100% corresponding to the treatment dose of 54 Gy. Inlet shows the magnified ipsilateral lung DVH

Variations of the normal lung volumes receiving high to low doses of 30 Gy to 5 Gy (i.e., *V*_*30Gy*_ to *V*_*5Gy*_) are shown in Fig. 1. Largest lung sparing was observed in seven out of ten plans at 60% PIL, with up to 23.2% and 19.9% reduction in *V*_*30Gy*_ to *V*_*20Gy*_ (*p* < 0.05), and at 70% PIL eight and nine out of ten plans produced better *V*_*30Gy*_ and *V*_*20Gy*_ (*p* < 0.05) compared to the reference plans at 85% PIL, respectively. The absolute changes of the mean *V*_*30Gy*_ and *V*_*20Gy*_ were however negligible, amounting to 0.9% and 1.4% for 60% PIL plans, and 0.9% to 1.3% for 70% PIL plans, respectively. For the low dose lung volume (V_*10Gy*_ and V_5Gy_), three plans showed lowest V_*10Gy*_ and *V*_*5Gy*_ at the 60% PIL while six and five plans showed lowest *V*_*10Gy*_ and *V*_*5Gy*_ at the 70% PIL, respectively. Figure 2 shows *V*_*54Gy*_ and the mean dose to the normal lung included in the PTV (i.e., PTV minus GTV). Results of dose constraint parameters of other OARs were given in Table 1. Statistical tests showed significant differences between the reference 85% and other PILs mainly for dose metrics of target volumes and normal lung. The potential of PIL–VMAT to increase the tumor dose while keeping the critical organ doses was illustrated in Fig. 3.

**Table 1:** Summary of the mean and one standard deviation of the dosimetric parameters obtained with PTV–based prescription

	85% PIL	80% PIL	70% PIL	60% PIL
GTV *D*_*mean*_ (Gy)	62.4 ± 1.5	66.2 ± 1.0	^a^74.4 ± 1.1	^a^84.2 ± 2.3
PTV *D*_*mean*_ (Gy)	58.7 ± 0.9	60.6 ± 0.7	^a^63.9 ± 0.8	^a^67.3 ± 1.7
PTV *D*_*98%*_ (Gy)	53.6 ± 1.7	53.1 ± 0.2	^a^52.9 ± 0.2	^a^52.6 ± 0.4
Spinal cord *D*_*1%*_ (Gy)	9.1 ± 2.9	10.0 ± 3.9	9.7 ± 3.8	10.2 ± 3.5
Esophagus *D*_*1%*_ (Gy)	9.4 ± 3.6	9.6 ± 4.9	9.5 ± 5.2	9.6 ± 5.2
Bronchus *D*_*1%*_ (Gy)	13.6 ± 7.6	13.2 ± 7.6	12.6 ± 7.2	12.0 ± 7.2
Trachea *D*_*1%*_ (Gy)	5.3 ± 7.1	5.6 ± 7.4	5.1 ± 6.9	5.3 ± 7.3
Chest wall/Rib *V*_*30G*y_ (cc)	42.0 ± 35.7	34.7 ± 19.0	33.1 ± 22.1	30.2 ± 20.5
Heart *D*_*1%*_ (Gy)	10.7 ± 9.4	10.5 ± 9.3	10.0 ± 9.0	9.8 ± 8.7
PTV minus GTV D_50%_ (Gy)	57.6 ± 0.7	59.2 ± 0.4	61.4 ± 0.8	63.5 ± 1.2
PTV minus GTV *V*_*54G*y_ (%)	34.5 ± 16.4	34.3 ± 16.2	34.2 ± 16.2	34.0 ± 15.9
Normal Lung MLD (Gy)	5.0 ± 1.7	4.7 ± 1.4	4.6 ± 1.3	4.6 ± 1.4
Normal Lung *V*_*30G*y_ (%)	4.9 ± 2.8	4.5 ± 2.0	4.1 ± 1.9	^a^4.0 ± 1.8
Normal Lung *V*_*20G*y_ (%)	8.4 ± 3.5	7.4 ± 3.0	7.1 ± 2.9	^a^7.0 ± 2.9
Normal Lung *V*_*10G*y_ (%)	13.9 ± 4.9	13.0 ± 4.1	^a^12.6 ± 3.6	^a^12.5 ± 3.8
Normal Lung *V*_*5G*y_ (%)	19.5 ± 4.9	19.2 ± 4.4	18.9 ± 4.2	18.8 ± 4.2
MU	4717 ± 994	^a^ 4333 ± 868	4615 ± 1013	5011 ± 1031
nCI	1.08 ± 0.03	1.07 ± 0.02	1.07 ± 0.01	1.07 ± 0.01
*R*_*50%*_	6.11 ± 1.42	5.73 ± 1.67	5.11 ± 1.26	^a^5.00 ± 1.07

Abbreviations: *GTV* gross target volume, *PTV* planning target volume, *D*_*mean*_ mean dose, *D*_*x%*_ dose to *x* percent volume of the organ, *V*_*xGy*_ percent volume of the organ receiving at least *x* Gy, *MLD* mean lung dose, *MU* monitor unit, *nCI* target dose conformity index, *R*_*50%*_ dose gradient index, *PIL* prescription isodose line relative to the maximum dose. ^a^Significant difference from the reference 85% PIL

Considering dose received by the chest wall and ribs, the 60% PIL resulted in the lowest averaged *V*_*30Gy*_. However, the chest wall volume receiving dose ≥100% of the prescription dose is higher with the 60% PIL (*n* = 4) by 0.3 to 12.8 cm^3^ than with the 85% reference PIL in 4 lesions that abutted to the chest wall.

**Fig. 4:**
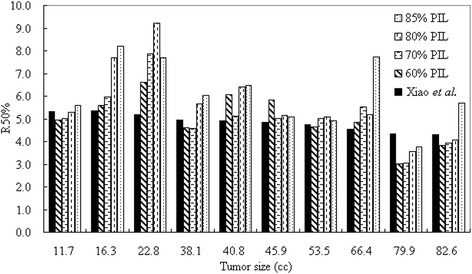
The dose gradient index *R*_*50%*_ for individual lesions sorted to size. Values of *R*_*50%*_ suggested by Xiao et al. [22] are also given

Both the target dose conformity (nCI) and the dose gradient (*R*_*50%*_) were found to be comparable between plans at different PILs (*p* > 0.05). Nonetheless, the *R*_*50%*_ dose constraint criterion, suggested by Xiao et al. [22], was met by the reference 85% PIL in only one patient, by 80% PIL in two patients, by 70% PIL in four patients and by 60% PIL in five patients (Fig. 4). The dose gradient was statistically better with 60% compared to the reference 85% PIL (*p* < 0.05).

### Correlation between PIL and LED

**Fig. 5:**
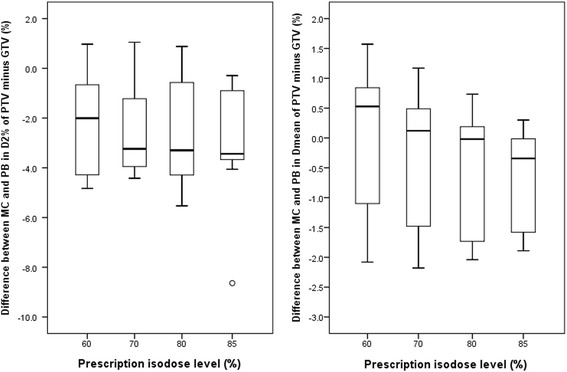
Dependence of Monte Carlo (MC) and pencil beam (PB) dose differences to the mean dose (*D*_mean_) and dose to 2% (*D*_2%_) of the lung tissue embedded in the planning target volume (PTV). Opened circles represent the individual patients and filled circles are the mean of all patients

Figure 5 shows the difference between the MC optimized and PB re-calculated plans in *D*_mean_ and *D*_98%_ of the PTV minus GTV border zone at varying PILs. The MC results predicted lower *D*_mean_ and *D*_98%_ than PB, and the differences increased with decreasing PILs, indicating increasing magnitude of LED. It is noteworthy thought that significant dependence of PIL was observed only for *D*_mean_ in the border zone.

### GTV–based prescription with varying PIL

**Fig. 6:**
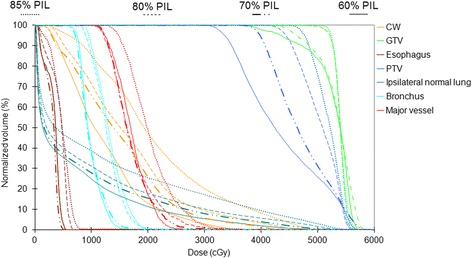
Dose–volume histograms of the same patient in Fig. 4, resulted from re–prescribing to the GTV mean dose, i.e. GTV–based prescription

The effect of inverse–optimization with GTV–based prescription concepts was clearly demonstrated in Fig. 6. After re–normalizing the prescription such that 50% of the GTV received 54 Gy, the peripheral dose of the PTV in terms of *D*_*95%*_ remained reasonably high, ranging from 33.5 to 36.1 Gy at 60% IDL, and 38.3 to 39.8 Gy at 70% IDL, respectively. Normal tissue doses were further decreased with decreasing PIL (Table 2). When compared with the reference 85% PIL plan employing PTV–based prescription, significant decreases of all normal tissue doses were achieved with 60% and 70% PIL by GTV–based prescription.

**Table 2:** Summary of the mean and one standard deviation of the dosimetric parameters obtained with GTV–based prescription

	85% PIL	80% PIL	70% PIL	60% PIL
GTV *D*_*mean*_ (Gy)	54.0 ± 0.1	53.9 ± 0.1	53.6 ± 0.2	54.0 ± 1.2
PTV *D*_*mean*_ (Gy)	50.7 ± 0.6	49.3 ± 0.7	46.1 ± 0.9	42.9 ± 1.1
PTV *D*_*98%*_ (Gy)	45.9 ± 1.2	43.2 ± 0.8	38.2 ± 0.5	33.6 ± 1.1
PTV *D*_*95%*_ (Gy)	46.7 ± 1.1	43.9 ± 0.7	39.0 ± 0.4	34.4 ± 0.9
Spinal cord *D*_*1%*_ (Gy)	7.9 ± 2.6	8.1 ± 3.2	7.0 ± 2.7	^a^6.5 ± 2.2
Esophagus *D*_*1%*_ (Gy)	8.2 ± 3.0	7.8 ± 4.1	^a^6.9 ± 3.8	^a^6.1 ± 3.3
Bronchus *D*_*1%*_ (Gy)	11.8 ± 6.6	10.9 ± 6.2	^a^9.1 ± 5.2	^a^7.6 ± 4.5
Trachea *D*_*1%*_ (Gy)	4.6 ± 6.1	4.5 ± 6.1	^a^3.7 ± 5.0	^a^3.4 ± 4.6
Chest wall/Rib *V*_*30G*y_ (cc)	29.8 ± 27.3	18.1 ± 16.3	^a^12.4 ± 12.4	^a^6.3 ± 8.0
Heart *D*_*1%*_ (Gy)	9.2 ± 8.3	8.5 ± 7.5	7.2 ± 6.5	^a^6.3 ± 5.5
PTV minus GTV D_50%_ (Gy)	49.8 ± 0.8	48.2 ± 0.6	^a^44.3 ± 0.6	^a^40.5 ± 0.6
PTV minus GTV *V*_*54G*y_ (%)	1.0 ± 1.8	1.3 ± 1.5	^a^0.4 ± 0.5	^a^0.1 ± 0.1
Normal Lung MLD (Gy)	4.4 ± 1.6	4.0 ± 1.4	^a^3.3 ± 1.0	^a^2.9 ± 0.9
Normal Lung *V*_*30G*y_ (%)	4.0 ± 2.6	3.1 ± 1.6	^a^2.2 ± 1.2	^a^1.7 ± 0.9
Normal Lung *V*_*20G*y_ (%)	7.0 ± 3.6	5.9 ± 2.6	^a^4.7 ± 2.2	^a^3.7 ± 1.7
Normal Lung *V*_*10G*y_ (%)	12.8 ± 4.9	11.3 ± 3.9	^a^9.8 ± 3.5	^a^9.4 ± 4.5
Normal Lung *V*_*5G*y_ (%)	18.2 ± 5.0	17.2 ± 4.2	^a^15.6 ± 3.7	^a^14.6 ± 3.8

*PIL* prescription isodose line relative to the maximum dose, *GTV* gross target volume, *PTV* planning target volume, *Dmean* mean dose, *Dx* dose to *x* percent volume of the organ, *Vx* percent volume of the organ receiving at least *x* Gy, *MU* monitor unit, *nCI* target dose conformity index, *R*_*50%*_ dose gradient index. ^a^Significant difference from the reference 85% PIL based on PTV *D*_95%_ prescription

## Discussion

This study demonstrated the technical feasibility of optimizing the PIL through its implicit relation with the lateral electronic disequilibrium (LED) to increase the dose gradient outside the target and hence improving the overall dosimetric quality in VMAT–based SBRT planning. This implicit relation was initially investigated in terms of the PTV dose differences between the PB and the MC dose calculation results for DCA–based lung SBRT [3]. This study followed the same methodology to understand the hypothesized LED origin of the dosimetric improvement. However, following the suggestion by Dish et al. [1] that the LED phenomenon can also be exploited in inverse optimization, our results were the first to show the dependence of PIL on LED for VMAT–type lung SBRT.

Using a reverse operation to re–calculate the type–B MC optimized dose plans with the type–A PB algorithms, as opposed to Zheng et al. [3] who re–calculated the PB–optimized plans by the MC algorithms, we found significant increase of (negative) dose differences of mean dose in the low density lung tissue embedded in the PTV (i.e., PTV minus GTV) with decreasing PIL plans indicative of increased magnitude of LED at lower PIL. The net negative dose difference can be explained by the increased fluence using type–B algorithms at 60% PIL in striving to compensate the LED deficiency at the low density tissue dominant field edge. The increased fluence led to increased overall dose deposition that was assumed by the type–A PB algorithms. The *D*_*2%*_ of the PTV border zone also showed similar trends of increasing (negative) dose differences with decreasing PIL despite insignificant statistical differences.

Unlike forward planning with DCA techniques, the advantage of inversely optimized VMAT is that the optimal MLC aperture relative to the tumor size can be solved through an intuitive adjustment of optimization parameters that are directly related to the clinical goals. Given the set of clinical goals, the optimizer would implicitly determine the optimal extent of LED adjusting for the photon beam energy as well as the variation of lung density between patients, avoiding the manual iteration of changing mostly the isotropic MLC margin to arrive at the desired PIL level, thus improving the planning efficiency. The direct incorporation of Monte Carlo dose engines further ensured that the dose distribution was robust against dosimetric errors caused by LED. Furthermore, this optimization approach is quite simple as we aimed at optimization of the PIL by adjusting the maximum and minimum dose (volume) to the target and the constraints of several dose controlling shell structures around the PTV.

Using this inverse optimization approach, the optimal PIL showing the most rapid dose falloff, which is significantly system design and planning technique dependent, was found to be between 60% and 70%. These results were consistent with previous reports by Oku et al. [4] showing best plan at 60% PIL. Despite the theoretical benefits of lower PIL than 60%, the associated toxicity profiles and local control remain largely unknown because it produced “hot spot” in the target beyond the acceptable range by most trial protocol [20, 21, 27] and therefore very limited clinical data are available. With the optimized PILs at 60% to 70% in present study, the GTV mean dose almost doubled in comparison to the reference 85% PIL without increasing lung dose, although the dose between the PTV and GTV was on average higher at 70% and 60% PIL by 6% and 10%, respectively. Considering this fact, it may be of genuine concern that the normal tissues embedded in this region, i.e. the non–tumorous margin region where motion and system inaccuracies are compensated for, receive higher doses. Given the treatment prescription of 54 Gy in 3 fractions delivered in 2 weeks for this study, 95% of the PTV will receive at least a biological effective dose (BED) of 151 Gy_10_, which is likely high enough to sterilize not just the tumor cells but also all other normal tissue cells as well. Yet, the increased chance of developing high grade radiation pneumonitis or fibrosis inside this small volume between GTV and PTV may be small as incidents of radiation pneumonitis or fibrosis are generally correlated with mean lung dose or low dose lung volume which were not increased at the lower 70–60% PIL [28]. Such assumption also has support from the recent DEGRO guidelines published by the German SBRT working group for early stage NSCLC which recommended a maximum dose of 150% to the PTV (i.e., ~ 65% PIL) based on the clinical evidences from a large–scale multi–center study [9].

Furthermore, this study clearly demonstrated that besides lung doses at optimized PIL as low as 60% doses to other serial OARs such as esophagus, heart, bronchus and trachea, major vessels, and spinal cord did not increase even for central tumors because the optimizer would automatically determine the set of anisotropic MLC margins variable with the gantry angle to achieve the specified dose constraints. This is generally impractical and labor–intensive in forward planning with DCA techniques. One of the exceptions could be tumors having the PTV overlapped with the chest walls. Nevertheless, this problem can be partly addressed by imposing more stringent dose–volume constraints on the chest wall and rib structures to push away the high dose. Also, the plan quality metric of the target dose conformity showed no statistical differences between different PILs. The dose gradient index was better for plans with 60% PIL than 80 and 85% PIL (*p* < 0.05) and was comparable between 60 and 70% PIL plans. The monitor units were found to increase with decreasing PIL in a linear manner likely resulting in minimally increased treatment time.

The additional advantage that comes along with optimized PIL at ~ 60 to 70% is the potential of further margin reduction. In this study, the mid–ventilation PTV was based on the van Herk’s margin recipe and calculated assuming a reference 85% PIL. The theoretical margin may be decreased by 1 to 2 mm from 85 to 60% PIL for our patient cohort whose observed motion was up to 2 cm, mainly due to the smaller *β* value of the inverse cumulative standard normal distributions at the prescribed PTV minimum dose level [19]. This margin reduction, although small, may leverage the dosimetric benefits of inverse optimization in VMAT even further, though we acknowledge that further studies are required in this regard and a discussion if the van Herk’s margin recipe can be used for inhomogeneous dose distributions is beyond the scope of this work.

Clinically, a BED of 100 Gy_10_, assuming an *α / β* ratio of 10 Gy, has been universally recognized as the approximate threshold dose to achieve adequate local control in early stage NSCLC, however, only based on a prescription to the PTV periphery [7, 29, 30] and with unclear PIL. On the other hand, Guckenberger et al. [7] recently hinted at lower PIL (< 80%) being significantly superior in local control in their data (86.8% vs. 69.1%, *p* = 0.005). Recently, the same retrospective large–scale multi–center study have reported on local control being also significantly dependent of the maximum isocenter dose [9, 10] which strongly supports our investigation of decreasing the PIL through inverse VMAT optimization and with that increasing the GTV doses. Following that concept and going one step further, a PTV prescription dose of 3 × 18 Gy at 85% PIL (151 Gy_10_ to PTV D_95%_ and *D*_*max*_ = 198 Gy_10_, reference dose level in our study) may very likely be reduced to 3 × 14 Gy at 60% PIL (101 Gy_10_ to PTV D_95%_ and *D*_*max*_ = 233 Gy_10_), resulting in substantial reductions in dose to OARs and even possibly in an increase in tumor control probability (TCP).

Based on this idea, this study further investigated the potential of significant dose de–escalation for SBRT by making best use of the physics derivable from the LED phenomenon. When we prescribed the dose in a way that GTV *D*_*50%*_ equals 54 Gy (BED = 151 Gy_10_) for all PIL with the aim to keep the TCP constant, the resulting BED in the PTV *D*_*95%*_ ranged from 71.0 to 79.5 Gy_10_, and 87.1 to 92.7 Gy_10_ at 60% and 70% PIL, respectively. While such PTV doses may seem low in comparison to previous publications, a recent investigation of PTV prescription dose reduction with constant high GTV mean doses found high local control for lung tumors even with low PTV prescription doses (PTV D_95%_ BED ≥89.7 Gy_10_ vs. < 89.7 Gy_10_, hazard ratio 0.077, confidence interval 0.012–0.503, *p* = 0.001,) [13]. Hence, we may hypothesize that 3 × 18 Gy prescribed to the mean GTV dose at 60 to 70% PIL may still result in high LC (> 90% [13]) while substantially reduce the dose to the lungs and other OARs.

In practice, prescription based on the GTV mean dose can be optimized for different combinations of dose fractionation schedule and PIL. For example, 54 Gy GTV mean dose could be delivered in 4 fractions at 60% PIL, producing PTV *D*_*95%*_ of 79.5 Gy_10_ that is roughly equivalent to 48 Gy in 4 fractions prescribed to the isocenter as commonly practiced in Japan [27]. For dose fractionation schedules with more fractions, 54 Gy GTV mean dose could be delivered in 5 fractions, but at slightly higher PIL at ~ 67%, producing PTV *D*_*95%*_ of 66.2 Gy_10_ equivalent to 50 Gy in 5 fractions reported by Aoki et al. [11] Alternatively, the prescription can also be re–normalized to higher median GTV dose than 54 Gy to achieve the desired PTV *D*_*95%*_ covered by the optimal PIL. Table 3 is provided to predict the 3–year local control rate from different dose schedules and prescription methods reported in the literature and in this study.

**Table 3:** Summary of the biological effective dose (BED) to 95% of the planning target volume (PTV *D*_*95%*_) and the 3–year local control (LC) rates in the literature

	Prescription	Isodose level at PTV edge	Dose algorithm	Total dose	BED (Gy_10_) in 3 fractions	BED (Gy_10_) in 4 fractions	BED (Gy_10_) in 5 fractions	3-years LC
Aoki et al. [11]	Isocenter	80%	Type-A	50			66.2	95.0%
Onishi et al. [12]								95.0%
Yoshitaki et al. [31]	Isocenter	80%	Type-A	48		62.3		90.0%
Shibamoto et al. [32]								87.0%
Ricardi et al. [33]	PTV edge	80%	Type-A	45	99.0			87.8%
Hassbeek et al. [34]	PTV edge	80%	Type-A	60 60	145.7		108.6	89.3% 89.3%
This study	GTV mean dose	70%	Type-B	54	89.2	76.7	69.1	
		60%	Type-B		74.3	64.4	58.4	

Note: BED_10_ (*α / β* = 10 Gy) was calculated after adjusting the physical prescription dose by a factor of 0.88 for type–A algorithm with respect to type–B Monte Carlo algorithm according to Ref [37]

Nevertheless, it is worthwhile to note that the concept of GTV–based prescription for lung SBRT and outcomes for such method are exclusively limited to robotic SBRT so far [13, 15], although the data is part of and fits nicely to recent TCP modeling [9, 10]. Clinical implementation of the proposed dose de–escalation approach by optimized PIL at ~ 60% with other techniques such as VMAT must be taken with great cautions, and further clinical studies are warranted to validate its efficacy and safety. It is admitted that this study did not fully address the implementation issues of GTV–based prescription. Future studies would be required to develop a link of the GTV prescription with the conventional concept of PTV prescription isodose line, the dose encompassing level of the PTV and their interactions with other patient–dependent / treatment technique–specific factors such as tumor motion range, PTV definition, etc. Further limitations to this study come from the limited number of presented cases as other parameter such as lesion location, volume and dimensions or density of the lesions itself could not have been statistically investigated.

## Conclusions

Optimizing the Monte Carlo calculated prescription isodose level for VMAT has the obvious advantage of improving the dosimetric quality of lung SBRT treatment plans and offers the possibility to achieve dose de–escalation. Further clinical investigation of gross tumor volume based dose prescription and optimal prescription isodose levels are warranted.

## Abbreviations

3DCT: Three–dimensional computed tomography
4DCT: Four–dimensional computed tomography
BED: Biological effective dose
DVH: Dose–volume–histogram
GTV: Gross target volume
LED: Lateral electronic disequilibrium
MC: Monte Carlo
MidV: Mid–ventilation
MLC: Multi–leaf collimator
MLD: Mean lung dose
MU: Monitor units
NSCLC: Non–small cell lung carcinoma
OAR: Organ–at–risks
PB: Pencil beam
PIL: Prescription isodose line
PTV: Planning target volume
SBRT: Stereotactic body radiotherapy
TCP: Tumor control probability
VMAT: Volumetric–modulated arc radiotherapy
